# Probiotic LB101 alleviates dry eye in mice by suppressing matrix metalloproteinase-9 expression through the regulation of gut microbiota-involved NF-κB signaling

**DOI:** 10.1371/journal.pone.0303423

**Published:** 2024-06-17

**Authors:** Xiaoyang Ma, Yoon-Jung Shin, Soo-Won Yun, Seok Won Jang, Seung-Won Han, Dong-Hyun Kim

**Affiliations:** 1 Neurobiota Research Center, College of Pharmacy, Kyung Hee University, Seoul, Korea; 2 PB Department, NVP Healthcare, Inc., Suwon, Korea; Arizona State University, UNITED STATES

## Abstract

Tear matrix metalloproteinase (MMP)-9 is an inflammatory signal in patients with dry eye (DE). In the present study, to understand the action mechanism of probiotic LB101 (*Lactobacillus plantarum* NK151 and *Bifidobacterium bifidum* NK175 [4:1] mix) against DE, we investigated its effect on tear amount and inflammatory marker expression levels in mice with unilateral exorbital lacrimal gland excision/atropine-benzalkonium chloride application (EB) or fecal microbiota transplantation from mice with EB (eFMT). Oral gavage of LB101 increased EB-suppressed tear amount and decreased EB-induced blinking number. Furthermore, LB101 decreased EB-induced TNF-α, IL-1β, and MMP-9 expression, TNF-α^+^ and NF-κB^+^CD11c^+^ cell populations, and edema in the conjunctiva, while EB-suppressed IL-10 and occludin expression increased. LB101 also decreased EB-induced TNF-α and IL-1β expression and NF-κB^+^CD11c^+^ cell population in the colon. eFMT also decreased tear amount and increased blinking number in the transplanted mice. eFMT increased TNF-α, IL-1β, and MMP-9 expression and TNF-α^+^ and NF-κB^+^CD11c^+^ cell populations in the conjunctiva and TNF-α and IL-1β expression and NF-κB^+^CD11c^+^ cell populations in the colon. Oral gavage of LB101 increased eFMT-suppressed tear amount and decreased eFMT-induced blinking number. Furthermore, LB101 decreased TNF-α, IL-1β, and MMP-9 expression, TNF-α^+^ and NF-κB^+^CD11c^+^ cell populations, and edema in the conjunctiva and TNF-α and IL-1β expression and NF-κB^+^CD11c^+^ cell population in the colon, while eFMT-suppressed IL-10 and occludin expression decreased. Furthermore, LB101 increased eFMT-suppressed *Muribaculaceae*, *Prevotellaceae*, and *Lactobacillaceae* populations in the gut microbiota, while eFMT-induced *Bacteroidaceae* population decreased. These findings suggest that DE may cause gut dysbiosis, which may be a risk factor for DE, and LB101 may alleviate DE with gut inflammation by suppressing the expression of MMP-9 and proinflammatory cytokines TNF-α and IL-1β with the regulation of gut microbiota-involved NF-κB signaling.

## Introduction

It is estimated that 5 to 30% of the world’s population suffers from dry eye (DE) [1, 2]. DE symptoms vary from mild discomfort to severe ocular fatigue and pain. DE is steadily increasing due to the popularization of smartphones, personal computers, air conditioning, and soft contact lenses [2, 3]. DE induces corneal epithelial and conjunctival damages through inflammation, which increases tumor necrosis factor (TNF)-α and matrix metalloproteinase (MMP)-9 expression in the ocular and conjunctival surface [4–6]. DE-induced long-term inflammation causes discomfort, visual disturbance, and tear film instability.

The desiccating stress increases the secretion of TNF-α and MMP-9 in the corneal epithelium and conjunctiva, which increase corneal irregularity and permeability due to the degradation/suppression of corneal and conjunctival tight junction proteins [7]. The excessive expression of TNF-α increases the expression and secretion of MMP-9 through NF-κB activation [8–10]. Therefore, MMP-9 inhibitors or their contained artificial tears have been used for DE therapy [6]. However, the long-lasting application of these medications frequently result in adverse reactions such as ocular hyperemia, bacterial infection, ocular hypertension, glaucoma, and cataract.

Gut dysbiosis is closely associated with many systemic disorders, including DE [11–14]. Patients with DE or Sjögren’s syndrome have an imbalanced gut microbiota composition compared to healthy volunteers [11, 15, 16]. Exposure to desiccating stress and restraint stress causes gut dysbiosis with DE in mice [17]. Unilateral exorbital lacrimal gland excision and the application of atropine/benzalkonium chloride (EB) also causes gut dysbiosis with DE in mice [18]. However, fecal microbiota transplantation (FMT) from healthy mice alleviates DE-like symptoms in germ-free CD25 KO mice [19]. Oral administration of a bifidobacterial mixture alleviates tear secretion in patients with DE [20]. Oral administration of *Lactobacillus plantarum* NK151, *Bifidobacterium bifidum* NK175, or their mix (LB101), which suppress the TNF-α to IL-10 expression ratio in macrophages, increase tear secretion and decrease keratitis and conjunctivitis in mice [18]. Nevertheless, there are few studies on the ameliorating action mechanism of probiotics against DE.

In the present study, we investigated the effect of LB101 on tear amount and conjunctivitis in EB mice or mice with their fecal microbiota transplantation.

## Materials and methods

### Materials

Phenol red-impregnated cotton threads were purchased from Zone Quick (Showa Yakuhin Kako Co., Ltd, Tokyo, Japan). A MRS medium was purchased from BD (Franklin Lakes, NJ, cat BD288130). Transgalactosylated oligosaccharide-mupirocin lithium salt agar plates (TOS-MU) were purchased from MB cell (Seoul, Korea, cat MB-T0892).

### Preparation of LB101

To select the anti-inflammatory probiotics, we investigated whether human fecal bacteria, which were isolated from human feces and deposited in Neurobiota Research Center, Kyung Hee University, could inhibit the TNF-α to IL-10 expression ratio in LPS-stimulated macrophages [18]. Of tested bacteria, NK151 and NK175 potently inhibited it. They were identified to be *Lactobacillus plantarum* and *Bifidobacterium bifidum*, based on gram staining, 16S rRNA gene sequencing, whole genome sequencing [18], and API kit analyses (S1 Table in S1 File). They were deposited in Korean Culture Center of Microorganisms (NK151, KCCM12783P; and NK175, KCCM12784P). They were cultured in general media for probiotics such as MRS broth and centrifuged at 5000 *g* and 4°C for 20 min [18]. Collected cells were freeze-dried. The viable number of freeze-dried NK151 and NK175 strains were determined using MRS and TOS-MU agars, respectively, by plating and incubating under an anaerobic condition. Four parts live NK151 to one part live NK175 (4:1) were mixed, named LB101, and suspended in sterilized distilled water for in vivo experiments.

### Animals

C57BL/6 mice (male, 18–21 g, 6 weeks old) were obtained from Orientbio Co. ltd. (Seongnam-shi, Korea) and acclimatized for 7 days before experiments. Mice were maintained in plastic cages with a 5-cm-raised wire floor under the controlled condition (temperature, 20–22°C, humidity, 50 ± 10%; light/dark cycle, 12 h). Mice were fed with water and a standard chow diet ad libitum.

All animal experiments were ethically approved by the Committee for the Care and Use of Laboratory Animals in the University (IACC, KHUASP(SE)-19-199) and were performed according to the Ethical Policies and Guidelines of Kyung Hee University for Laboratory Animals Care and Use. This study additionally adheres to standards articulated in the ARRIVE guidelines [21]

### Preparation of mice with dry eye

Frist, mice with EB-induced DE were prepared, as previously reported [18]. Briefly, mice were separated into four groups (NC, Sham, DE, LB101). Each group consisted of six mice. In DE and LB101 groups, right exorbital lacrimal gland (ELG) was excised under isoflurane anesthetization and, from next day, one drop of 1% atropine/0.1% benzalkonium chloride (AB) solution was put in the cornea of the right eye twice a day for 5 days. In LB101 group, LB101 (5×10^8^ CFU/mouse/day) were orally gavaged once a day for 2 weeks (6 days in one week). Sham group was operated without the resection of ELG and treated with saline instead of AB. NC (normal control group), Sham, and DE were treated with the vehicle.

Second, mice with FMT-induced DE were prepared by transplanting the fecal microbiota (suspended in saline) of mice with EB-induced DE. Briefly, mice were separated into three groups (NC, hFMT, eFMT, LB101). Each group consisted of six mice. The fecal microbiota (1×10^9^ CFU, suspended in 0.1 mL of saline) of mice with EB-induced DE were orally transplanted in eFMT and LB101 groups once a day for 5 days. The suspension of fecal microbiota (1×10^9^ CFU, suspended in 0.1 mL saline) from normal control mice were orally transplanted in hFMT mice once a day for 5 days. Thereafter, in LB101 group, LB101 (5×10^8^ CFU/mouse/day) were orally gavaged once a day for 2 weeks (6 days in one week). NC (normal control group), hFMT, and eFMT mice were treated with vehicle instead of LB101. Bacterial counts in the fecal microbiota suspension was measured using GAM agar plate by plating and incubating in an anaerobic condition, as previously reported [22].

Mice were euthanized by exposure to CO_2_ in a chamber, followed by cervical dislocation. Cornea, conjunctiva, and colon tissues were collected and stored at −80°C for the assay of biochemical markers.

For the immunofluorescence assay, mice were transcardiacally perfused with 4% paraformaldehyde, as previously reported [18]. Cornea/conjunctiva and colon tissues were post-fixed with 4% paraformaldehyde for 4 h, cytoprotected in 30% sucrose solution, frozen, and sectioned using a cryostat.

### Tear amount measurement

Tear amounts were measured 18 h after the final treatment with LB101 or vehicle [18]. Phenol red-impregnated cotton threads were placed in the lateral canthus for 30 s. Tear amounts were indicated as the tear fluid-infiltrated color-changed thread length (cm).

### Eye-blinking counting

Mouse faces were recorded for 3 min using a digital camera and the eye-blinking number was counted [18].

### Enzyme-linked immunosorbent assay (ELISA) and immunoblotting

Conjunctiva and colon tissues were homogenized and lysed in RIPA buffer (150 mM sodium chloride, 1% sodium deoxycholate, 1% Triton X-100, 0.1% SDS, 50 mM Tris–HCl, 2 mM EDTA, pH 7.5) containing a phosphatase inhibitor cocktail (Roche, Basel, Switzerland: cat #4906837001) and centrifuged at 10,000 *g* and 4°C for 20 min, as previously reported [18].

For the ELISA, the expression levels of TNF-α (R&D system, Minneapolis: cat# MN DY410), IL-1β (R&D system: cat #DY401), MMP-9 (B&D system: cat #MMPT90), and myeloperoxidase (R&D system: cat #DY3667) were measured in the supernatant, using their ELISA kits.

For the immunoblotting, the supernatants of conjunctiva homogenates were separated by 10% SDS-polyacrylamide gel electrophoresis, transferred to polyvinylidenedifluoride membrane (Millipore, Burlington, MA), then hybridized overnight at 4℃ with antibodies for occludin (1:1000, Abcam: #ab222691) or β-actin (1:2000, Santa Cruz Biotechnology, Dallas, TX: cat # sc-47778), and washed 3 times with PBS containing 0.1% Tween 20 (PBST) for 10 min, and incubated with horseradish peroxidase-conjugated secondary antibody horseradish peroxidase-conjugated secondary antibody (1:5000, Santa Cruz Biotechnology), and visualized with a luminescent image analyzer (LAS-4000; Fujifilm, Tokyo, Japan) [23]. The band density was quantified using Image Lab software (Bio-Rad, Hercules, CA).

### Immunofluorescence assay

Immunofluorescence assay was performed, as previously reported [18, 24]. Briefly, the sections of eye with conjunctiva were washed with phosphate-buffered saline, blocked with normal serum, incubated with antibodies for TNF-α (1:1000, Abcam: cat #ab183218), NF-κB (p-p65, 1:100, Cell Signaling Technology: cat # 3033S), and/or CD11c (1:100, Abcam: cat #ab11029) overnight, and treated with the secondary antibody conjugated with Alexa Fluor 488 (1:200, Invitrogen) or Alexa Fluor 594 (1:200, Invitrogen) for 2 h. Nuclei were stained with 4′,6-diamidino-2-phenylindole, dilactate (Sigma, cat #F6057). The sections were observed using a confocal microscope.

### Quantitative polymerase chain reaction

To measure bacterial populations in the gut microbiota, bacterial DNA was purified from the fresh feces of mice using QIAamp Fast DNA stool mini kit (Qiagen, Hilden, Germany: cat #51604) [25]. qPCR analysis for gut bacterial genes was performed using SYBER premix Ex Taq II (TaKaRa, Shiga, Japan: cat #RR820). Thermal cycling condition: initial denaturation, 95°C for 30 sec; denaturation, 95℃ for 5 sec, annealing, 63°C for 30 sec; extension, 72°C for 30 sec; cycle number, 40. The normalized expression of assayed genes, with respect to bacterial rRNA, was computed for all samples using the Microsoft Excel data spreadsheet. The primers used in qPCR analysis, which were previously developed [26–30], are shown in S2 Table in S1 File.

### Statistical analysis

All data are given as mean ± standard deviation (SD) and analyzed using GraphPad Prism 9 (GraphPad Software Inc., San Diego, CA). The significance was analyzed using one-way ANOVA Dunnett’s multiple comparisons test (p < 0.05) (S3 Table in S1 File).

## Results

### Effect of LB101 on tear amount and conjunctivitis in mice with EB-induced DE

**Fig 1:**
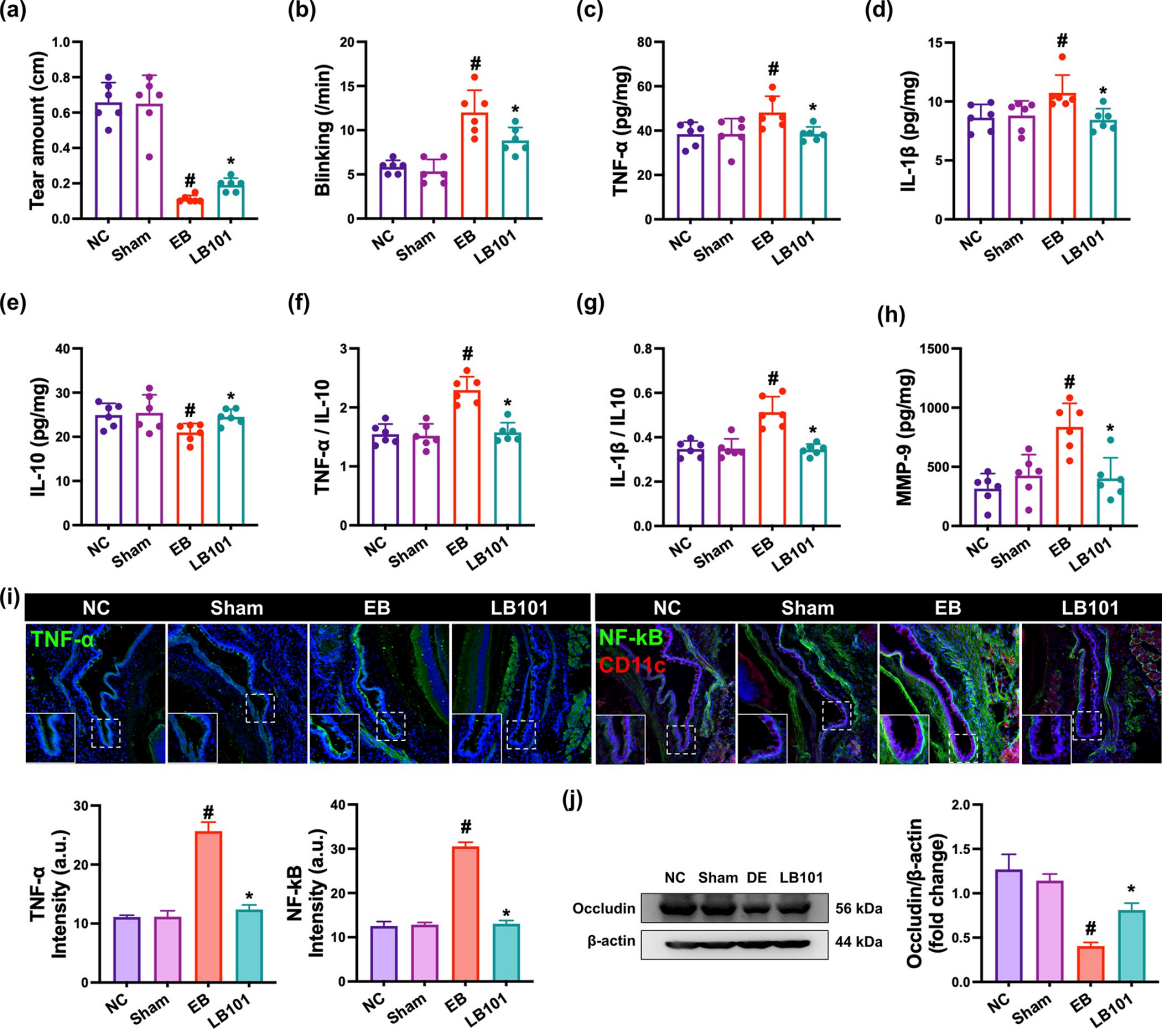
Effect of LB101 on EB-induced dry eye in mice. (a) Effect on tear amount. (b) Effect on blinking number. Effect on the IL-1β (c), TNF-α (d), and IL-10 expression (e), IL-1β to IL-10 expression ratio (f), TNF-α to IL-10 expression ratio (g), and MMP-9 expression (h), assessed by ELISA. (i) Effect on the TNF-α^+^ and NF-κB^+^CD1c^+^ cell populations in the conjunctiva, assessed by immunofluorescence assay. (j) Effect on occludin expression in the conjunctiva, assessed by immunoblotting. NC, vehicle; vehicle in normal control group; Sham, vehicle in mice operated without the resection of ELG; DE, vehicle in mice with the resection of ELG; LB101, probiotic LB101 (5×10^8^ CFU/mouse/day) in mice with the resection of ELG. Data values are described as mean ± SD (*n* = 6). ^#^p<0.05 vs. sham. *p<0.05 vs. EB.

To confirm the effect of LB101 against DE, we investigated the effect of LB101 in mice with EB-induced DE (Fig 1A and 1B). EB treatment significantly decreased tear amount to 16.7% of sham mice (F_3,20_ = 50.8, p<0.001) and increased the blinking number to 237.3% of sham mice (F_3,20_ = 20.73, p<0.001), as previously reported [18]. Oral gavage of LB101 significantly increased EB-suppressed tear amount to 28.1% of sham mice and decreased EB-induced blinking number to 166.7% of sham mice, as previously reported [18].

Next, we examined the effect of LB101 on the expression of proinflammatory and anti-inflammatory cytokines in the conjunctiva of EB-exposed mice (Fig 1C–1I). Exposure to EB increased the expression of proinflammatory cytokines IL-1β and TNF-α and population of TNF-α^+^ cells and decreased the expression of anti-inflammatory cytokine IL-10 and occludin compared to normal control mice. Resultingly, EB increased the expression ratio of TNF-α or IL-1β to IL-10, as previously reported [18]. Oral gavage of LB101 significantly decreased the expression of proinflammatory cytokines IL-1β and TNF-α and population of TNF-α^+^ cells in EB-treated mice, while the expression of anti-inflammatory cytokine IL-10 decreased.

Exposure to EB also increased MMP-9 expression and NF-κB^+^CD11c^+^ cell population in the conjunctiva compared to normal control mice, while occludin expression was decreased (Fig 1I and 1J). In particular, EB-induced NF-κB-positive cell population was positively proportional to TNF-α-positive cell population and TNF-α expression in hippocampus of EB-exposed mice. However, oral gavage of LB101 significantly decreased EB-induced MMP-9 expression and NF-κB^+^CD11c^+^ cell population and increased EB-suppressed occludin expression.

### Effect of LB101 on gastrointestinal inflammation in mice with EB-induced DE

**Fig 2:**
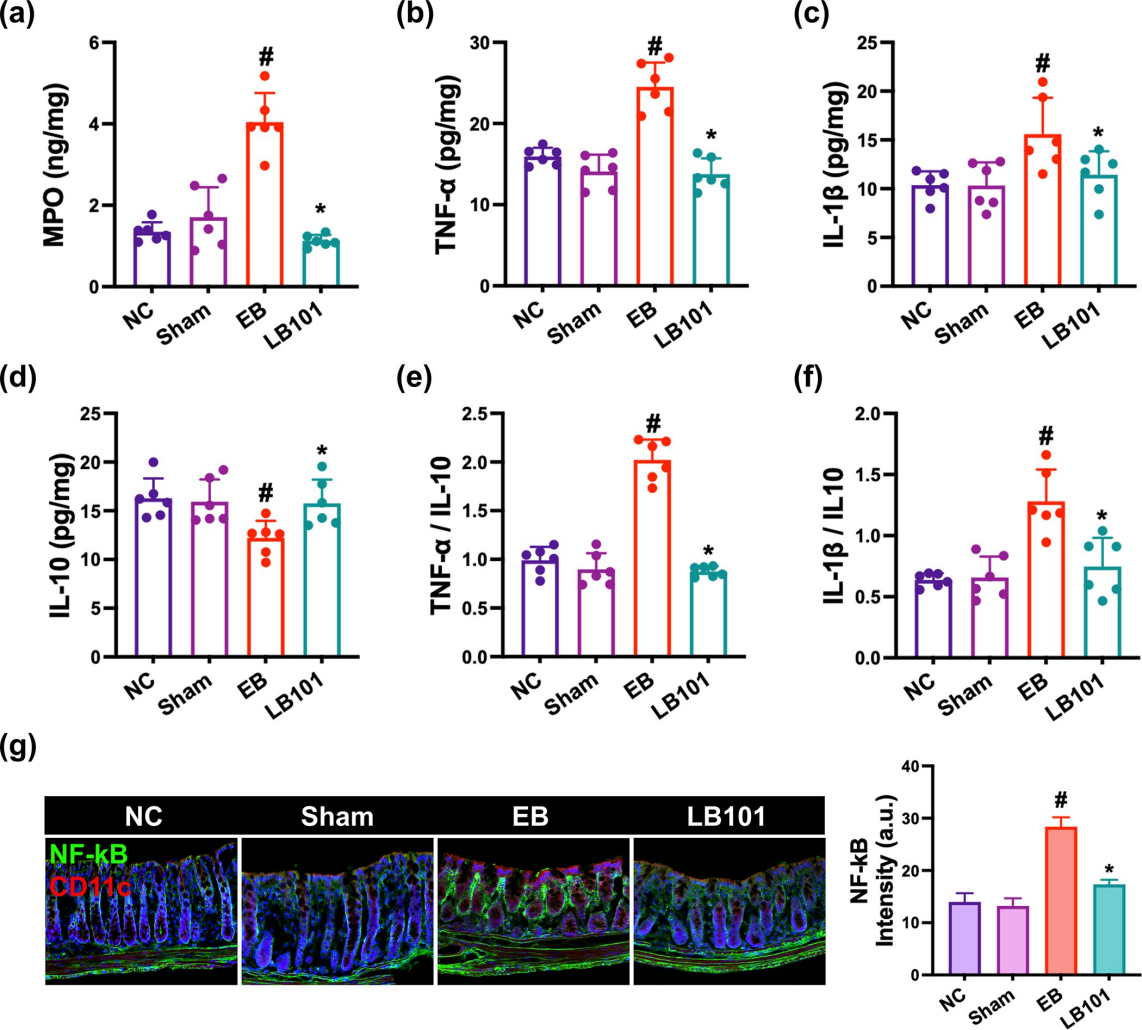
Effect of LB101 on EB-induced colitis in mice. (a) Effect on myeloperoxidase (MPO, a), IL-1β (b), TNF-α (c), and IL-10 expression (d) in the colon, assessed by ELISA. Effect on IL-1β to IL-10 expression ratio (e) and TNF-α to IL-10 expression ratio (f). (g) Effect on the NF-κB^+^CD1c^+^ cell populations in the colon, assessed by immunofluorescence assay. NC, vehicle; vehicle in normal control group; Sham, vehicle in mice operated without the resection of ELG; DE, vehicle in mice with the resection of ELG; LB101, probiotic LB101 (5×10^8^ CFU/mouse/day) in mice with the resection of ELG. Data values are described as mean ± SD (*n* = 6). ^#^p<0.05 vs. sham. *p<0.05 vs. EB.

Next, the effect of LB101 on the myeloperoxidase, IL-1β, IL-10, and TNF-α expression and NF-κB^+^CD11c^+^ cell population was investigated in the colon of mice with EB-induced DE (Fig 2, S1 Fig in S1 File). EB treatment weakly, but not significantly, caused bodyweight loss and colon shortening and edema. Nevertheless, EB treatment significantly increased myeloperoxidase, TNF-α, and IL-1β expression and NF-κB^+^CD11c^+^ cell population, as previously reported [18]. EB-induced NF-κB-positive cell population was positively proportional to TNF-α expression in colon of EB-exposed mice. However, oral gavage of LB101 decreased myeloperoxidase, TNF-α, and IL-1β expression and NF-κB^+^CD11c^+^ cell population in mice with EB-induced DE, while IL-10 expression increased.

### Effect of LB101 on tear amount, conjunctivitis, colitis, and gut dysbiosis in mice transplanted with fecal microbiota of mice with EB-induced DE

**Fig 3:**
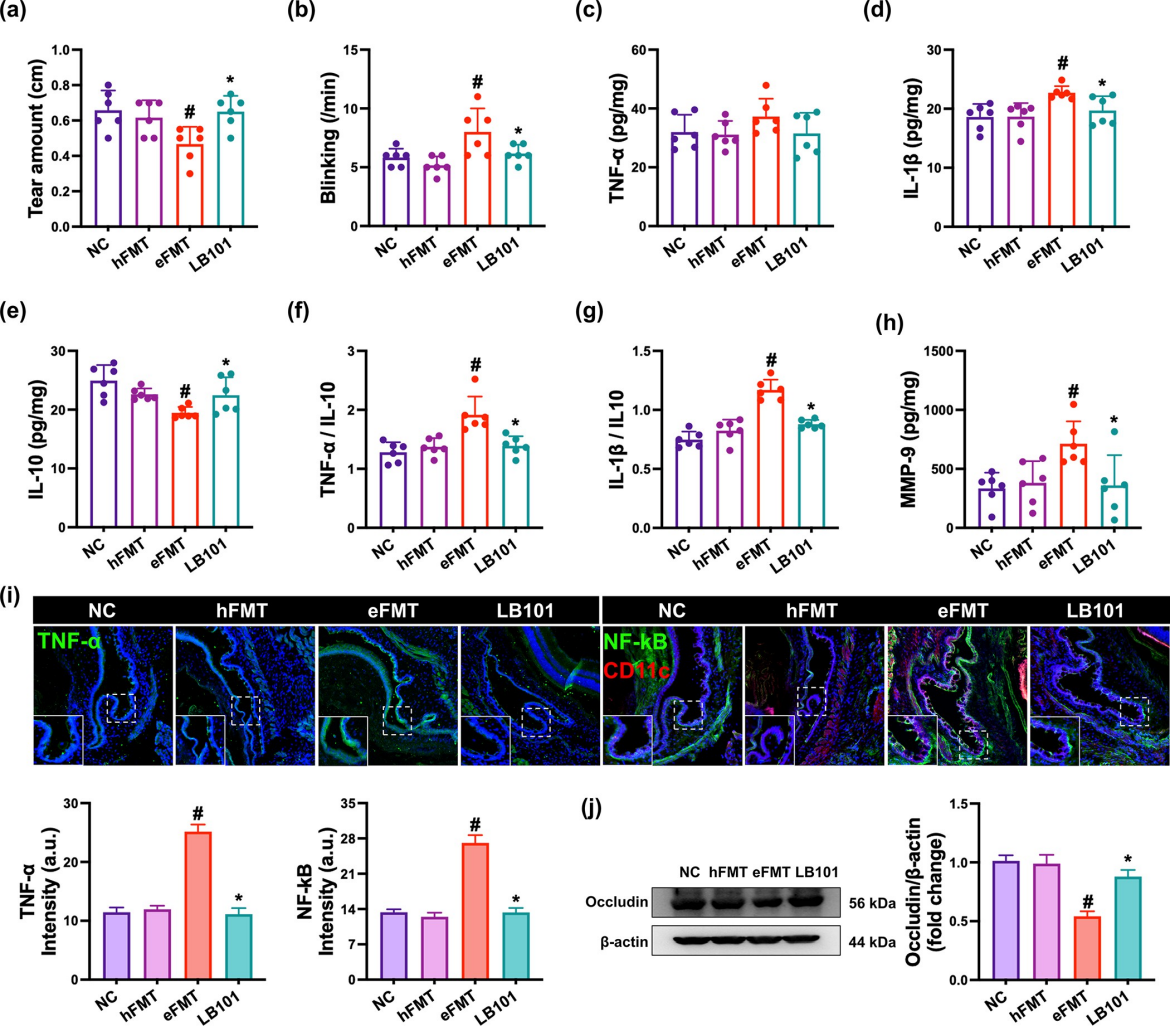
Effect of LB101 on eFMT-induced dry eye in mice. (a) Effect on tear amount. (b) Effect on blinking number. Effect on the IL-1β (c), TNF-α (d), and IL-10 expression (e), IL-1β to IL-10 expression ratio (f), TNF-α to IL-10 expression ratio (g), and MMP-9 expression (h), assessed by ELISA. (i) Effect on the TNF-α^+^ and NF-κB^+^CD1c^+^ cell populations in the conjunctiva, assessed by immunofluorescence assay. (j) Effect on occludin expression in the conjunctiva, assessed by immunoblotting. NC, vehicle; vehicle in normal control group; hFMT, vehicle in mice transplanted with healthy mouse gut microbiota; eFMT, vehicle in mice with eFMT-induced dry eye; LB101, probiotic LB101 (5×10^8^ CFU/mouse/day) in mice with eFMT-induced dry eye. Data values are described as mean ± SD (*n* = 6). ^#^p<0.05 vs. hFMT. *p<0.05 vs. eFMT.

Exposure to EB causes gut dysbiosis with DE in mice [18]. When the correlation-based network analysis for the previously reported data [18] related to the gut microbiota composition of mice treated with or without EB was performed, EB treatment increased the *Bacteroidaceae* population, which was positively correlated with tear secretion (amount), but decreased *Lactobacillaceae* and *Muribaculaceae* populations, which were negatively correlated with tear secretion (S1 Fig in S1 File). Therefore, to understand whether EB-induced gut dysbiosis could cause DE, we transplanted the fecal microbiota from mice with EB-induced DE or healthy control mice in specific pathogen-free mice and investigated its effect on the occurrence of DE (Fig 3). Although the fecal microbiota transplantation from healthy mice (hFMT) did not affect tear amount and blinking number, the fecal microbiota transplantation from mice with EB-induced DE (eFMT) decreased tear amount to 72.9% of hFMT (F_3,20_ = 4.8, p = 0.011) and increased blinking number to 155.1% of hFMT (F_3,20_ = 6.1, p = 0.004). Furthermore, the eFMT increased MMP-9, IL-1β, and TNF-α expression and TNF^+^ and NF-κB^+^CD11c^+^ cell populations and decreased IL-10 and occludin expression compared to normal control mice. The eFMT also increased TNF-α or IL-1β to IL-10 expression ratio. Moreover, eFMT-induced NF-κB-positive cell population was positively proportional to TNF-α-positive cell population and TNF-α expression in eFMT-exposed mice, as in EB-exposed mice.

However, oral gavage of LB101 recovered eFMT-suppressed tear amount to 104.4% of hFMT and decreased eFMT-induced blinking number to 119.4% of hFMT. Furthermore, LB101 decreased eFMT-induced MMP-9, IL-1β, and TNF-α expression and TNF^+^ and NF-κB^+^CD11c^+^ cell populations. LB101 also decreased eFMT-induced TNF-α or IL-1β to IL-10 expression ratio. However, LB101 increased eFMT-suppressed IL-10 and occludin expression.

**Fig 4:**
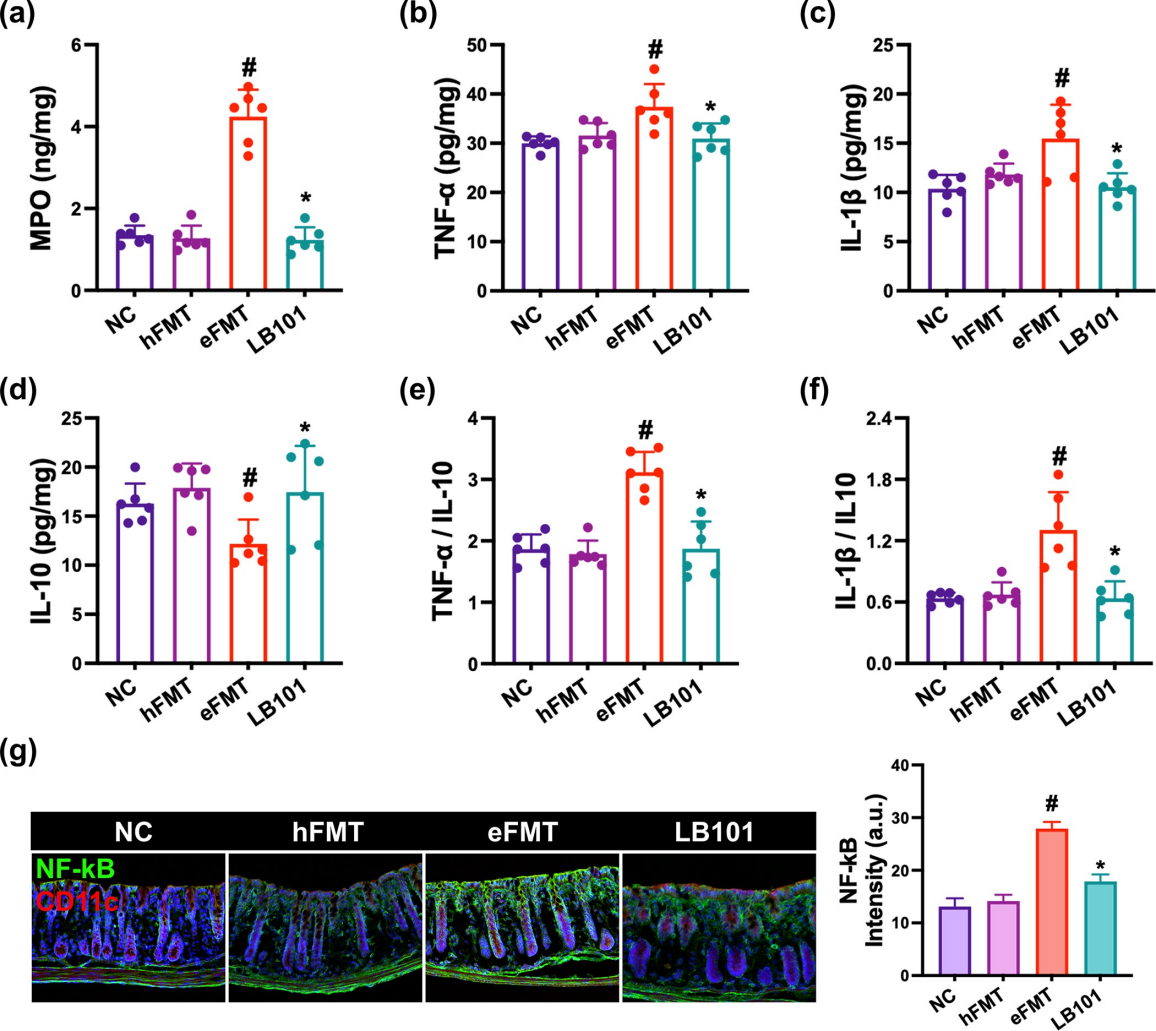
Effect of LB101 on eFMT-induced colitis in mice. (a) Effect on myeloperoxidase (MPO, a), IL-1β (b), TNF-α (c), and IL-10 expression (d) in the colon, assessed by ELISA. Effect on IL-1β to IL-10 expression ratio (e) and TNF-α to IL-10 expression ratio (f). (g) Effect on the NF-κB^+^CD1c^+^ cell populations in the colon, assessed by immunofluorescence assay. NC, vehicle; vehicle in normal control group; hFMT, vehicle in mice transplanted with healthy mouse gut microbiota; eFMT, vehicle in mice with eFMT-induced dry eye; LB101, probiotic LB101 (5×10^8^ CFU/mouse/day) in mice with eFMT-induced dry eye. Data values are described as mean ± SD (*n* = 6). ^#^p<0.05 vs. hFMT. *p<0.05 vs. eFMT.

Next, we investigated the effect of LB101 on myeloperoxidase, IL-1β, IL-10, and TNF-α expression and NF-κB^+^CD11c^+^ cell population in the colon of mice with eFMT-induced DE (Fig 4). Although eFMT treatment significantly increased myeloperoxidase, TNF-α, and IL-1β expression and NF-κB^+^CD11c^+^ cell population, IL-10 expression decreased. EB treatment weakly, but not significantly, caused bodyweight loss and colon shortening and edema. Oral gavage of LB101 decreased eFMT-induced myeloperoxidase, TNF-α, and IL-1β expression and NF-κB^+^CD11c^+^ cell population and increased eFMT-suppressed IL-10 expression. Resultingly, LB101 decreased eFMT-induced TNF-α or IL-1β to IL-10 expression ratio. eFMT-induced NF-κB-positive cell population was positively proportional to TNF-α expression in the colon of eFMT-exposed mice, as in EB-exposed mice.

**Fig 5:**
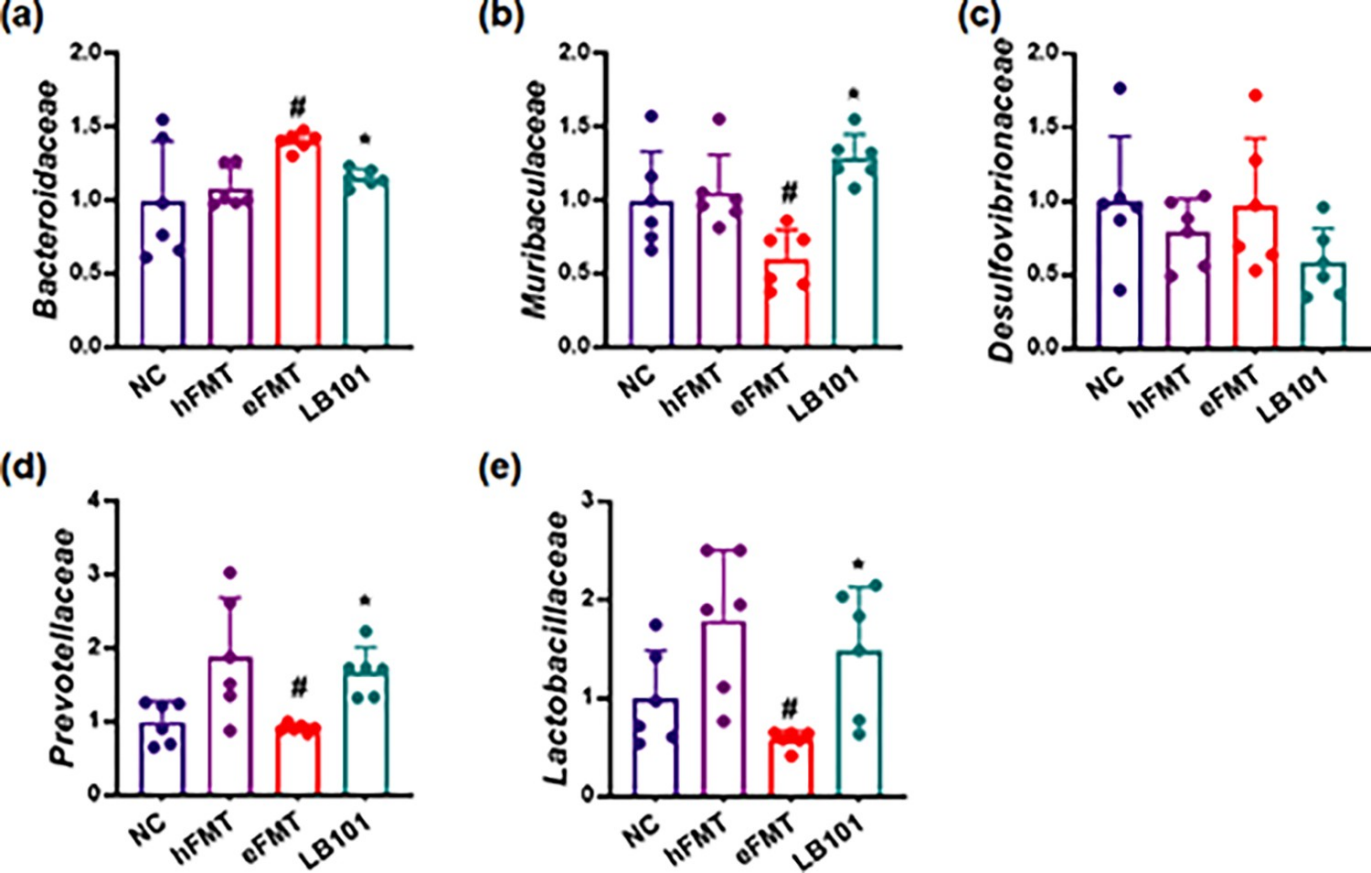
Effect on LB101 on fecal *Bacterioidaceae*, *Muribaculaceae*, *Desulfovibrionaceae*, *Prevotellaceae*, and *Lactobacillaceae* populations in mice with eFMT-induced DE. Data values are described as mean ± SD (*n* = 6). ^#^p<0.05 vs. hFMT. *p<0.05 vs. eFMT.

We measured the effect of LB101 on the populations of gut microbiota *Bacteroidaceae*, *Lactobacillaceae*, *Muribaculaceae*, *Desulfovibrionaceae*, and *Prevotellaceae* in mice with eFMT-induced DE (Fig 5). eFMT significantly increased *Bacteroidaceae* population and decreased *Muribaculaceae*, *Lactobacillaceae*, and *Prevotellaceae* populations. However, LB101 increased eFMT-suppressed induced *Muribaculaceae*, *Lactobacillaceae*, and *Prevotellaceae* populations, while eFMT-increased *Bacteroidaceae* population was decreased.

## Discussion

Apical epithelial cells in the ocular surface produce various mucins including MUC1, MUC4, MUC5, and MUC16 to sustain smoothness and hydration [31]. Lacrimal glands also secrete tear with antimicrobial factors such as immunoglobulin A, lactoferrin, lysozyme, and defensins [32, 33]. However, exposure to desiccating stress in the corneal epithelium increases the tear osmolarity and induces the expression of inflammatory cytokines such as TNF-α and gelatinases such as MMP2 and MMP9 in the cornea and conjunctiva by activating NF-κB signaling [7, 34–36]. Thus, desiccation increases the concentrations of inflammatory cytokines and gelatinases in tears, resulting in keratitis and conjunctivitis. In particular, MMP-2 and MMP-9 cause the disruption of corneal barrier with the digestion of mucins, resulting in an increase in corneal irregularity and permeability and conjunctivitis with the desquamation of the corneal epithelium [7, 37]. Tear secretion is inversely correlated with the MMP-9 concentration in the tear [7, 38]. Cyclosporine A, an immunosuppressant, increases tear fluid and decreases MMP-9 expression by inhibiting inflammation in the conjunctiva [4, 34]. Based on these findings, it is suggested that MMP-9 may be a potential target for the treatment of DE.

In the present study, EB treatment decreased tear amount and increased blinking number, conjunctival edema, TNF-α expression, and TNF^+^ cell population, as previously reported [18]. Furthermore, EB treatment increased MMP-9 expression and NF-κB^+^CD11c^+^ cell populations in the conjunctiva. These results suggest EB can induce TNF-α and IL-1β expression in the cornea and conjunctiva by activating NF-κB signaling, which induces MMP-9 expression in immune cells including neutrophils. Moreover, we found that EB decreased IL-10 and occludin expression in the conjunctiva. The MMP9 impairs corneal epithelia. These results suggest that EB-induced MMP9 can suppress the expression levels of tight junction proteins including occludin, resulting in DE with keratitis and conjunctivitis.

Exposure to EB increased the expression of proinflammatory cytokines including TNF-α and population of NF-κB^+^CD11c^+^ cells in the colon, as previously reported [18]. Yun et al. reported that EB caused gut dysbiosis with colitis in mice: it increased the *Bacteroidaceae* population and decreased *Muribaculaceae*, *Prevotellaceae*, and *Lactobacillaceae* populations in the gut microbiota, assessed by 16S rRNA gene sequencing [18]. Moon et al., Choi et al., and Schaefer et al. reported that DE was closely connected with gut microbiota fluctuation in patient and mice [11, 15, 39, 40]. These findings suggest that EB-induced DE may be closely associated with the occurrence of gut inflammation and dysbiosis.

Based on these findings, we transplanted the fecal microbiota from mice with EB-induced DE and investigated DE-related symptoms. eFMT (FMT from mice with EB-induced DE) decreased tear amount and increased blinking number and conjunctival edema in the transplanted mice, like mice with EB. eFMT also increased conjunctival MMP-9 and TNF-α expression and conjunctival TNF^+^ and NF-κB^+^CD11c^+^ cell populations, while IL-10 and occludin (tight junction protein) expression decreased. However, FMT from healthy mice did not affect tear amount and blinking number, and conjunctival edema in the transplanted mice. Furthermore, eFMT also increased TNF-α and IL-1β expression and NF-κB^+^CD11c^+^ cell population in the colon, resulting in colitis. eFMT also fluctuated gut microbiota: it increased the *Bacteroidaceae* population and decreased *Muribaculaceae*, *Prevotellaceae*, and *Lactobacillaceae* populations in the gut microbiota, as previously reported in mice with EB-induced DE [18]. These results suggest that DE may be closely associated with gut dysbiosis, which can induce MMP-9, TNF-α, and IL-1β expression and suppress tight junction protein occludin (a tight junction protein) expression in the conjunctiva through NF-κB activation, resulting in DE with conjunctivitis. Moreover, eFMT may be beneficial for the preparation of experimental animal with DE.

LB101 increased tear amount and decreased blinking in mice with EB- or eFMT-induced DE. Furthermore, LB101 suppressed EB- or eFMT-induced MMP-9, TNF-α, and IL-1β expression and increased EB- or eFMT-suppressed IL-10 and occludin expression in the conjunctiva. LB101 also decreased EB- or eFMT-induced TNF-α^+^ and NF-κB^+^CD11c^+^ cell population in the conjunctiva. These findings suggest that LB101 can alleviate DE and conjunctivitis by suppressing MMP-9 expression and inducing occludin expression with the regulation of NF-κB signaling.

LB101 decreased EB- or eFMT-induced TNF-α and IL-1β expression and NF-κB^+^CD11c^+^ population in the colon. LB101 increased eFMT-suppressed *Muribaculaceae*, *Prevotellaceae*, and *Lactobacillaceae* populations and decreased the eFMT-induced *Bacteroidaceae* population in the gut microbiota. Yun et al. also reported that LB101 decreased EB-induced *Bacteroidaceae*, *Akkemansiaceae*, and AC160630_f populations and increased EB-suppressed *Lactobacillaceae* and *Muribaculaceae* populations in the gut microbiota [18]. These results suggest that LB101 can alleviate EB- or eFMT-induced gut inflammation and dysbiosis by suppressing NF-κB signaling with the regulation of gut microbiota.

## Conclusions

Exposure to EB caused DE including conjunctiva, colitis, and gut dysbiosis. The FMT from mice with EB-induced DE (eFMT) reduced tear amount and induced conjunctiva, colitis, and gut dysbiosis in the transplanted mice. LB101 alleviated DE with colitis by suppressing MMP-9 expression and increasing tight junction protein with the regulation of gut microbiota-involved NF-κB signaling.

## Supporting information

S1 File This file contains tables and figures of additional data. [S1 Table. Differential characteristics of NK151 and NK175, based on API kits; S2 Table. Primers used for qPCR analysis; S3 Table. p values of Figs 1–4; S1 Fig. The relationship between gut microbiota and tear secretion (TS) in mice with EB-induced DE, assessed by Pearson correlation analysis].
(DOCX)

S1 Raw images This file contains all raw images of immunoblotting data. [Figs 1(J) and 3(J)].
(PDF)

S1 Data (XLSX)

S2 Data (XLSX)
