# Supplementary material for: Probiotic LB101 alleviates dry eye in mice by suppressing matrix metalloproteinase-9 expression through the regulation of gut microbiota-involved NF-κB signaling
Source: PLoS One. 2024 Jun 17;19(6):e0303423. doi: 10.1371/journal.pone.0303423 (PMC11182509; doi:10.1371/journal.pone.0303423)
Supplement: S1 File — [S1 Table. Differential characteristics of NK151 and NK175, based on API kits; S2 Table. Primers used for qPCR analysis; S3 Table. p values of Figs 1–4; S1 Fig. The relationship between gut microbiota and tear secretion (TS) in mice with EB-induced DE, assessed by Pearson correlation analysis]. (DOCX) [file pone.0303423.s001.docx]

**Supporting information [1]**

**Probiotic LB101 alleviates dry eye in mice by suppressing metalloproteinase-9 expression through the regulation of gut microbiota-involved NF-κB signaling**

S1 Table. Differential characteristics of NK151 and NK175, based on API kits

| API kit (50CHL) | NK151 | API kit (20A) | NK175 |
| --- | --- | --- | --- |
| - | - | L-tryptophan | - |
| Glycerol | + | Urea | - |
| Erythritol | - | D-glucose | + |
| D-arabinose | - | D-mannitol | - |
| L-arabinose | + | D-lactose | + |
| D-ribose | + | D-sucrose | - |
| D-xylose | - | D-maltose | + |
| L-xylose | - | Salicin | - |
| D-adonitol | - | D-xylose | + |
| Methyl-β-D-xylopyranoside | - | L-arabinose | - |
| D-galactose | + | Gelatin | - |
| D-glucose | + | Esculin | + |
| D-fructose | + | Glycerol | - |
| D-mannose | + | D-cellobiose | + |
| L-sorbose | - | D-mannose | + |
| L-rhamnose | - | D-melezitose | - |
| Dulcitol | - | D-raffinose | + |
| Inositol | - | D-sorbitol | - |
| Mannitol | + | D-rhamnose | - |
| Sorbitol | - | D-trehalose | - |
| α-methyl-D-mannoside | + | CAT | - |
| α-methly-D-glucoside | - | Spor | - |
| N-acetyl-glucosamine | + | Gram | + |
| Amygdalin | + | Cocci | - |
| Arbutin | + |  |  |
| Esculin | + |  |  |
| Salicin | + |  |  |
| Cellobiose | + |  |  |
| Maltose | + |  |  |
| Lactose | + |  |  |
| Melibiose | + |  |  |
| Sucrose | + |  |  |
| Trehalose | + |  |  |
| Inulin | - |  |  |
| Melezitose | + |  |  |
| Raffinose | + |  |  |
| Starch | - |  |  |
| Glycogen | - |  |  |
| Xylitol | - |  |  |
| Gentiobiose | + |  |  |
| D-turanose | + |  |  |
| D-lyxose | - |  |  |
| D-tagatose | - |  |  |
| D-fucose | - |  |  |
| L-fucose | - |  |  |
| D-arabitol | - |  |  |
| L-arabitol | - |  |  |
| gluconate | + |  |  |
| 2-keto-gluconate | - |  |  |
| 5-keto-gluconate | - |  |  |

S2 Table. Primers used for qPCR analysis

| Family |  | Primer sequence |
| --- | --- | --- |
| Bacteroidaceae | Forward | GAAGGTCCCCCACATTG |
|  | Reverse | CGCKACTTGGCTGGTTCAG |
| Lactobacillaceae | Forward | TGGAAACAGRTGCTAATACCG |
|  | Reverse | GTCCATTGTGGAAGATTCCC |
| Muribaculaceae | Forward | GAGAGTACCTGAAGAAAAAGC |
|  | Reverse | ACGCATTCCGCATACTTCT |
| Desulfovirionaceae | Forward | CCGTAGATATCTGGAGGAACATCAG |
|  | Reverse | ACATCTAGCATCCATCGTTTACAGC |
| Prevotellaceae | Forward | CCAGCCAAGTAGCGTGCA |
|  | Reverse | TGGACCTTCCGTATTACCGC |
| 16S rRNA gene | Forward | TCGTCGGCAGCGTCAGATGTGTATAAGAGACAGGTGCCAGCMGCCGCGGTAA |
|  | Reverse | GTCTCGTGGGCTCGGAGATGTGTATAAGAGACAGGGACTACHVGGGTWTCTAAT |

S3 Table. p values of figures 1, 2, 3, and 4.

| Figure 1 | | | |
| --- | --- | --- | --- |
|  | | NC&EB | EB&LB101 |
| (a) | Tear amount (cm) F (3, 20) = 50.80 | p=0.000 | p=0.0488 |
| (b) | Blinking (/min) F (3, 20) = 20.73 | p=0.000 | p=0.0140 |
| (c) | TNF-α (pg/mg) F (3, 20) = 4.014 | p=0.0266 | p=0.0270 |
| (d) | IL-1β (pg/mg) F (3, 20) = 4.442 | p=0.0212 | p=0.0119 |
| (e) | IL-10 (pg/mg) F (3, 20) = 3.147 | p=0.0237 | p=0.0403 |
| (f) | TNF-α / IL-10 F (3, 20) = 22.19 | p=0.000 | p=0.000 |
| (g) | IL-1β / IL10 F (3, 20) = 18.91 | p=0.000 | p=0.000 |
| (h) | MMP-9 (pg/mg) F (3, 20) = 11.03 | p=0.000 | p=0.0008 |
| (i) | TNF-α Intensity (a.u.) F (3, 20) = 328.9 | p=0.000 | p=0.000 |
|  | NF-kB Intensity (a.u.) F (3, 20) = 723.0 | p=0.000 | p=0.000 |
| (j) | occludin/β-actin intensity F (3, 20) = 110.8 | p=0.000 | p=0.000 |
|  |  |  |  |
| Figure 2 | | | |
|  | | NC&EB | EB&LB101 |
| (a) | MPO (ng/mg) F (3, 20) = 38.28 | p=0.000 | p=0.000 |
| (b) | TNF-α (pg/mg) F (3, 20) = 33.49 | p=0.000 | p=0.000 |
| (c) | IL-1β (pg/mg) F (3, 20) = 5.477 | p=0.0066 | p=0.0318 |
| (d) | IL-10 (pg/mg) F (3, 20) = 4.682 | p=0.0104 | p=0.0253 |
| (e) | TNF-α / IL-10 F (3, 20) = 78.51 | p=0.000 | p=0.000 |
| (f) | IL-1β / IL10 F (3, 20) = 14.24 | p=0.000 | p=0.0004 |
| (g) | NF-kB Intensity (a.u.) F (3, 20) = 167.9 | p=0.000 | p=0.000 |
|  |  |  |  |
| Figure 3 | | | |
|  | | NC&eFMT | eFMT&LB101 |
| (a) | Tear amount (cm) F (3, 20) = 4.8 | p=0.0111 | p=0.0125 |
| (b) | Blinking (/min) F (3, 20) = 6.1 | p=0.004 | p=0.0390 |
| (c) | TNF-α (pg/mg) F (3, 20) = 1.420 | p=0.3069 | p=0.2499 |
| (d) | IL-1β (pg/mg) F (3, 20) = 5.151 | p=0.0075 | p=0.0414 |
| (e) | IL-10 (pg/mg) F (3, 20) = 6.547 | p=0.0003 | p=0.0250 |
| (f) | TNF-α / IL-10 F (3, 20) = 11.48 | p=0.000 | p=0.0009 |
| (g) | IL-1β / IL10 F (3, 20) = 36.32 | p=0.000 | p=0.000 |
| (h) | MMP-9 (pg/mg) F (3, 20) = 4.957 | p=0.0087 | p=0.0146 |
| (i) | TNF-α Intensity (a.u.) F (3, 20) = 327.8 | p=0.000 | p=0.000 |
|  | NF-kB Intensity (a.u.) F (3, 20) = 335.0 | p=0.000 | p=0.000 |
| (j) | occludin/β-actin intensity F (3, 20) = 89.84 | p=0.000 | p=0.000 |
|  |  |  |  |
| Figure 4 | | | |
|  | | NC&eFMT | eFMT&LB101 |
| (a) | MPO (ng/mg) F (3, 20) = 77.80 | p=0.000 | p=0.000 |
| (b) | TNF-α (pg/mg) F (3, 20) = 6.912 | p=0.0015 | p=0.0052 |
| (c) | IL-1β (pg/mg) F (3, 20) = 7.983 | p=0.0010 | p=0.0014 |
| (d) | IL-10 (pg/mg) F (3, 20) = 4.212 | p=0.0334 | p=0.0081 |
| (e) | TNF-α / IL-10 F (3, 20) = 23.74 | p=0.000 | p=0.000 |
| (f) | IL-1β / IL10 F (3, 20) = 14.27 | p=0.000 | p=0.0004 |
| (g) | NF-kB Intensity (a.u.) F (3, 20) = 203.4 | p=0.000 | p=0.000 |


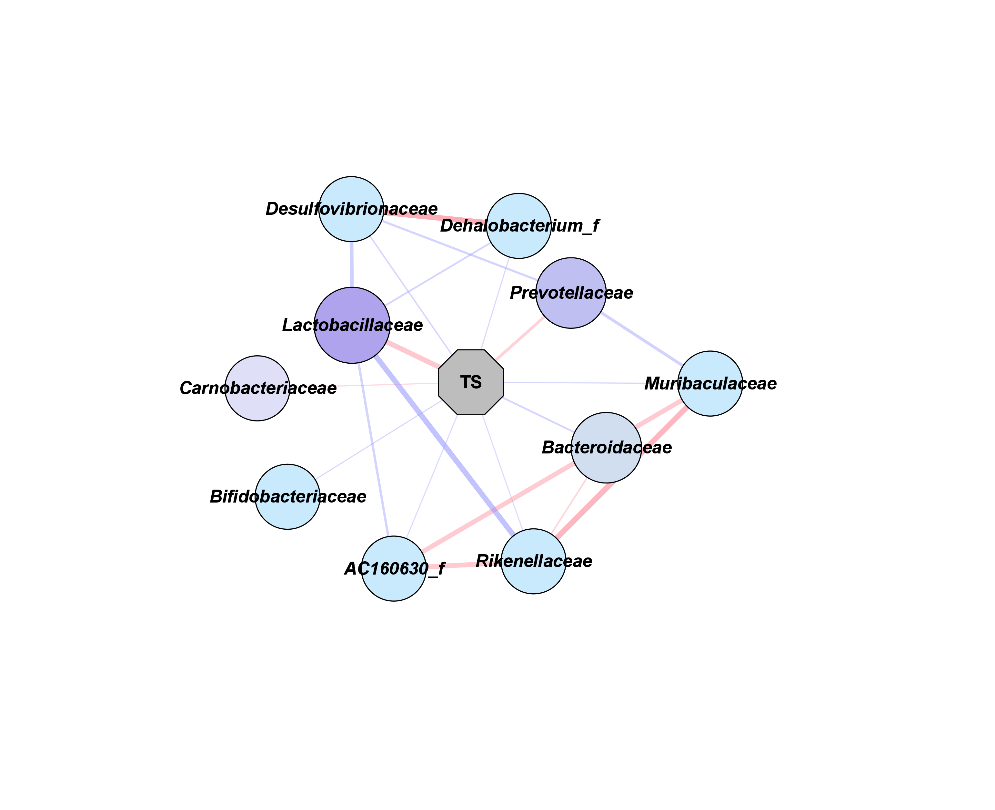


| Taxon Name | r | P (two-tailed) | P value summary | Significant (alpha = 0.05) |
| --- | --- | --- | --- | --- |
| *AC160630_f* | -0.2762 | 0.0767 | ns | No |
| *Bacteroidaceae* | -0.3221 | 0.0375 | * | Yes |
| *Bifidobacteriaceae* | -0.3020 | 0.0519 | ns | No |
| *Carnobacteriaceae* | 0.2785 | 0.0741 | ns | No |
| *Dehalobacterium_f* | -0.2955 | 0.0575 | ns | No |
| *Desulfovibrionaceae* | -0.3171 | 0.0408 | * | Yes |
| *Lactobacillaceae* | 0.4310 | 0.0044 | ** | Yes |
| *Muribaculaceae* | -0.3018 | 0.0521 | ns | No |
| *Prevotellaceae* | 0.3428 | 0.0262 | * | Yes |
| *Rikenellaceae* | -0.2649 | 0.0900 | ns | No |

S1 Fig. The relationship between gut microbiota and tear secretion (TS) in mice with EB-induced DE, assessed by Pearson correlation analysis. Data are indicated as mean ± SD (n = 6).
