# Supplementary material for: Probiotic LB101 alleviates dry eye in mice by suppressing matrix metalloproteinase-9 expression through the regulation of gut microbiota-involved NF-κB signaling
Source: PLoS One. 2024 Jun 17;19(6):e0303423. doi: 10.1371/journal.pone.0303423 (PMC11182509; doi:10.1371/journal.pone.0303423)
Supplement: S1 Raw images — [Figs 1(J) and 3(J)]. (PDF) [file pone.0303423.s002.pdf]

(A)

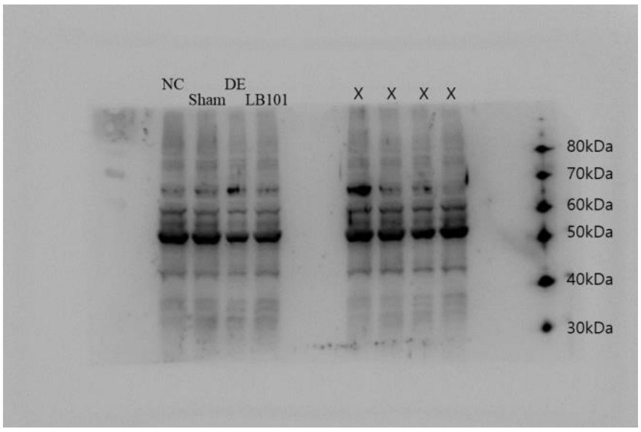

(B)

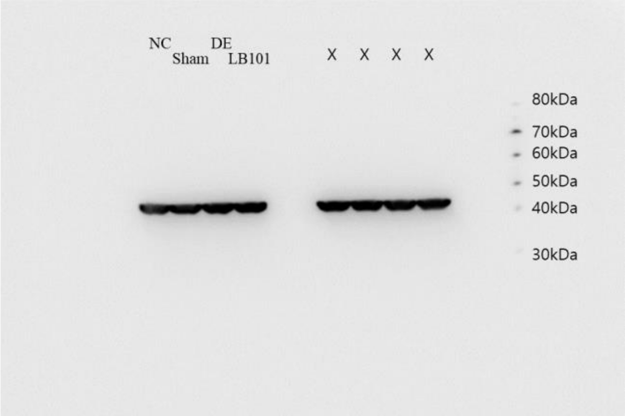

[Raw_images of Figure 1 (j) immunoblotting]

Effect of LB101 on occludin (A) and β-action (B) expression in the conjunctiva of mice with EB-induced dry eye. NC, vehicle; vehicle in normal control group; Sham, vehicle in mice operated without the resection of ELG; DE, vehicle in mice with the resection of ELG; LB101, probiotic LB101 (5×10^8 CFU/mouse/day) in mice with the resection of ELG.

(A)

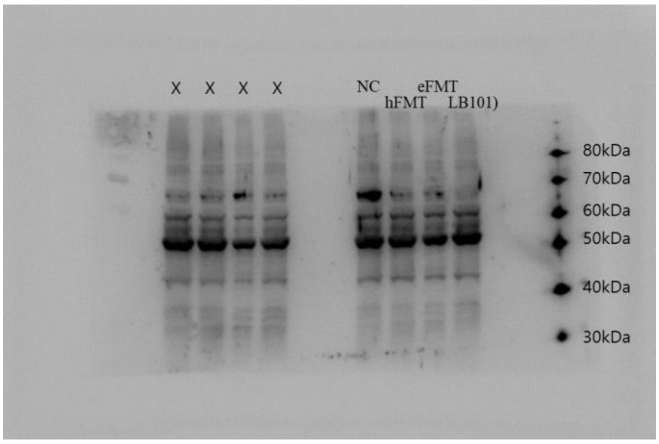

(B)

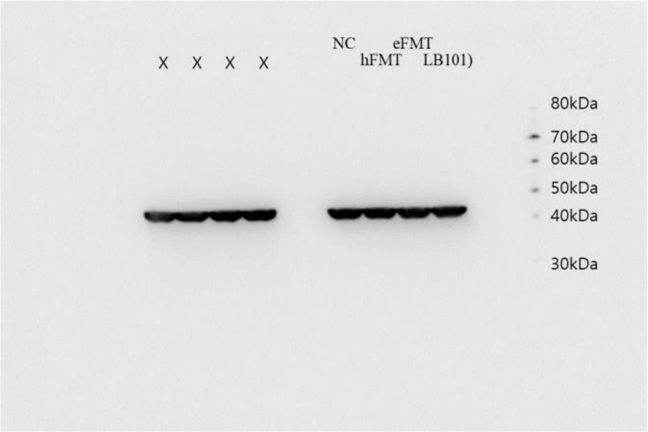

[Raw_images of Figure 3 (j) immunoblotting]

Effect of LB101 on occludin (A) and β-action (B) expression in the conjunctiva of mice with eFMT-induced dry eye. NC, vehicle; vehicle in normal control group; hFMT, vehicle in mice transplanted with healthy mouse gut microbiota; eFMT, vehicle in mice with eFMT-induced dry eye; LB101, probiotic LB101 (5×10^8 CFU/mouse/day) in mice with eFMT-induced dry eye.
